# Splicing dysregulation contributes to the pathogenicity of several *F9* exonic point variants

**DOI:** 10.1002/mgg3.840

**Published:** 2019-06-30

**Authors:** Upendra K. Katneni, Aaron Liss, David Holcomb, Nobuko H. Katagiri, Ryan Hunt, Haim Bar, Amra Ismail, Anton A. Komar, Chava Kimchi‐Sarfaty

**Affiliations:** ^1^ Hemostasis Branch, Division of Plasma Protein Therapeutics, Office of Tissues and Advanced Therapies, Center for Biologics Evaluation & Research US FDA Silver Spring Maryland; ^2^ Department of Statistics University of Connecticut Storrs Connecticut; ^3^ Department of Biological, Geological and Environmental Sciences, Center for Gene Regulation in Health and Disease Cleveland State University Cleveland Ohio

**Keywords:** hemophilia B, in silico splicing analysis, in vitro minigene assay, splicing dysregulation, synonymous variants

## Abstract

**Background:**

Pre‐mRNA splicing is a complex process requiring the identification of donor site, acceptor site, and branch point site with an adjacent polypyrimidine tract sequence. Splicing is regulated by splicing regulatory elements (SREs) with both enhancer and suppressor functions. Variants located in exonic regions can impact splicing through dysregulation of native splice sites, SREs, and cryptic splice site activation. While splicing dysregulation is considered primary disease‐inducing mechanism of synonymous variants, its contribution toward disease phenotype of non‐synonymous variants is underappreciated.

**Methods:**

In this study, we analyzed 415 disease‐causing and 120 neutral *F9* exonic point variants including both synonymous and non‐synonymous for their effect on splicing using a series of in silico splice site prediction tools, SRE prediction tools, and in vitro minigene assays.

**Results:**

The use of splice site and SRE prediction tools in tandem provided better prediction but were not always in agreement with the minigene assays. The net effect of splicing dysregulation caused by variants was context dependent. Minigene assays revealed that perturbed splicing can be found.

**Conclusion:**

Synonymous variants primarily cause disease phenotype via splicing dysregulation while additional mechanisms such as translation rate also play an important role. Splicing dysregulation is likely to contribute to the disease phenotype of several non‐synonymous variants.

## 1 INTRODUCTION

Pre‐mRNA splicing is the natural process of excision of noncoding introns and joining of exons (Caminsky, Mucaki, & Rogan, 2015). Splicing requires the detection of acceptor site (or 3′ splice site) and donor site (or 5′ splice site) located at the intron‐exon junctions and branch point site with adjacent polypyrimidine tract sequence located about 18–40 nucleotides upstream of acceptor site in intron by the spliceosome machinery (Cartegni, Chew, & Krainer, 2002). Splicing machinery primarily consists of five small nuclear ribonucleoproteins (snRNPs; U1, U2, U4, U5, and U6) and several other proteins (Will & Lührmann, 2001). The consensus sequence of donor site is 10 nucleotides long and extends from −3 in exon to +6 in intron with position 0 being the first nucleotide of intron. The consensus sequence of acceptor site is 28 nucleotides long extending from position −25 in intron to +2 in exon. Within these consensus sequences, for donor sites, nucleotides G and T are highly conserved at positions 0 and +1 and nucleotides A and G are predominant at positions −2 and −1. For acceptor sites, nucleotides A and G are highly conserved at positions −1 and 0 and nucleotide G is predominant at position +1 (Caminsky et al., 2015; Cartegni et al., 2002). The remaining positions in these consensus sequences are less stringent and the degree of consensus in these positions determines the strength of the natural splice sites and the ability of snRNPs to recognize them (Caminsky et al., 2015). In addition, 6–8 nucleotides long splicing regulatory elements (SREs) located in both exons and introns act as binding sites for splicing activator or repressor proteins and play an important role in regulation of splicing. Based on the location and their effect on splicing, SREs are classified as exonic splicing enhancers or silencers (ESEs or ESSs) and intronic splicing enhancers or silencers (ISEs or ISSs; Caminsky et al., 2015). Enhancer SREs and silencer SREs exert their action by acting as binding sites for serine‐arginine rich (SR) proteins and heterogeneous nuclear ribonucleoprotein (hnRNP), respectively (Chen, Kobayashi, & Helfman, 1999; Graveley, 2000).

The human *F9* gene (OMIM: 300746) is located on the long arm of X‐chromosome (Xq27.1–q27.2) and encodes a serine protease zymogen; coagulation factor IX (FIX; Anson et al., 1984; Rallapalli, Kemball‐Cook, Tuddenham, Gomez, & Perkins, 2013). *F9* gene spans about 34 kilobase pairs in length and is comprised of eight exons and seven introns (Anson et al., 1984). The primary *F9* transcript encodes a 461 amino acid (aa) long pre‐pro‐protein which is processed to yield a 415 aa mature FIX.

The partial or complete absence of FIX leads to the blood clotting disorder, hemophilia B (Bowen, 2002). Based on the level of FIX deficiency, hemophilia B is classified as severe (<1% of normal), moderate (1%–5% of normal), and mild (5%–40% of normal; White et al., 2001). Hemophilia B is primarily an inherited X‐linked recessive disorder caused by variants in *F9* gene. The current version of Center for Disease Control and Prevention's (CDC) Hemophilia B Mutation Project (CHBMP) database (f9‐CHBMP‐v5‐5‐5‐15) contains 1,131 *F9* variants (Li, Miller, Payne, & Craig Hooper, 2013). These *F9* variants are classified as missense variants (657, 58.1%), nonsense variants (91, 8%), intronic variants causing splice site changes (106, 9.4%), frameshift variants (182, 16.1%), large structural changes of >50 base pairs (bp; 33, 2.9%), small structural changes (23, 2%), promoter variants (25, 2.2%), 3′Untranslated region variants (4, 0.4%), and synonymous variants (10, 0.9%). Of these, missense variants and synonymous variants in exonic regions are point variants that involve single‐nucleotide substitution and make up majority (59%) of *F9* variants.

Synonymous variants do not alter the underlying aa sequence and are considered to primarily cause disease by altering splicing of pre‐mRNA transcript (Hunt, Simhadri, Iandoli, Sauna, & Kimchi‐Sarfaty, 2014). Missense (non‐synonymous) variants alter the aa sequence of the protein which is considered to be the main causative mechanism of the disease. However, recent studies revealed that significant proportion of these variants also affect splicing (Caminsky et al., 2015; Soukarieh et al., 2016; Sterne‐Weiler, Howard, Mort, Cooper, & Sanford, 2011), thus suggesting another, yet so far, underappreciated role of the altered splicing in the origin of the disease.

Synonymous and non‐synonymous point variants in exonic regions that alter sequences of splice sites and SREs dysregulate the splicing of pre‐mRNAs. Depending on the context of variant, splicing disruption by variants can lead to either exon skipping, intron inclusion, mis‐splicing, or leaky splicing (Caminsky et al., 2015). Ideally, the effect of variants on splicing is best characterized by analyzing the RNA transcripts isolated from patient's clinical sample. In the case of hemophilia B, *F9* is primarily expressed in liver and analysis of RNA transcripts from peripheral blood to assess splicing is not reliable (Green, Rowley, & Giannelli, 2003). Since it is not feasible to obtain primary hepatocytes, researchers in the field of hemophilia B have to rely on in silico prediction tools and in vitro assays to assess the impact of *F9* variants on pre‐mRNA splicing.

In this study, we employed a series of in silico splicing analysis tools in conjunction with in vitro minigene assays to assess the effect of *F9* exonic variants and neutral variants on pre‐mRNA splicing. Results from our study showed that splicing dysregulation contributes to disease phenotype of several *F9* non‐synonymous variants. In silico splice site and SRE prediction tools in tandem better predicted the splicing dysregulation caused by variants; however, outcome of splicing dysregulation seems to be influenced by several additional factors. In vitro minigene assay is an effective tool to assess the effect of splicing dysregulation when clinical samples are not available.

## 2 MATERIALS AND METHODS

### 2.1 Database construction

Disease‐associated exonic single‐nucleotide variants in *F9* gene were obtained from databases described by Hamasaki‐Katagiri et al. (Hamasaki‐Katagiri et al., 2013), the CDC Hemophilia B Mutation Project database (http://www.cdc.gov/ncbddd/hemophilia/champs.html; Li et al., 2013) and EAHAD FIX Gene Variant Database (http://www.factorix.org/; Rallapalli et al., 2013). *F9* neutral single‐nucleotide variants were extracted from NCBI dbSNP (https://www.ncbi.nlm.nih.gov/snp; Reference sequence: NM_000133.3; Sherry et al., 2001). Only unique single‐nucleotide variants in the open reading frame (ORF) for which both genetic and clinical characterization data are available were included in the database; insertions/deletions, frameshift variants, and nonsense variants were excluded. *F9* variant database generated based on these criteria included a total of 535 unique exonic point variants (Table 1; Table S1), of these, 415 are disease‐causing (409 non‐synonymous and six synonymous) and 120 are neutral (64 non‐synonymous and 56 synonymous). Variants of all three severities (mild, moderate, and severe) are collectively considered “disease‐associated” variants for the current study. The nomenclature for the variants in the database and manuscript was based on the recent guidelines for genetic variants in hemostasis (Goodeve, Reitsma, & McVey, 2011).

**Table 1:** Exonic distribution of *F9* disease‐causing and neutral variants analyzed in the study

Exon	Disease‐causing non‐synonymous variants	Disease‐causing synonymous variants	Neutral non‐synonymous variants	Neutral synonymous variants
1	7	1	10	3
2	59	1	13	4
3	8	0	0	2
4	50	0	2	4
5	37	2	3	7
6	34	2	10	6
7	33	0	6	7
8	181	0	20	23
Total	409	6	64	56

### 2.2 In silico analysis using splice site prediction tools

To study the effect of *F9* exonic disease‐causing and neutral variants on native splice site disruption and/or cryptic splice site creation/activation, we employed NNSplice, MaxEntScan (MES), SpliceSiteFinder‐like (SSF‐like), and Human Splicing Finder (HSF) splice site prediction tools that are included in the Alamut visual software (Interactive Biosoftware, Rouen, France. https://www.interactive-biosoftware.com/alamut-visual/). These tools employ different methods to predict splice sites: NNSPLICE employs two “neural networks” that were trained on consensus splice sites, while also considering dinucleotide frequencies due to the strong correlation between neighboring nucleotides in splice site consensus sequences (Reese, Eeckman, Kulp, & Haussler, 1997). MES scores sequences for splice sites by employing “maximum entropy principle” that takes into account adjacent and nonadjacent position dependencies in consensus splice sites (Yeo & Burge, 2004). SSF‐like and HSF employ “position weight matrices” constructed from aligned consensus sequences to weigh each nucleotide in a sequence and uses the sum of individual nucleotide's weights to derive its potential splice site strength (Desmet et al., 2009; Shapiro & Senapathy, 1987). To assess the splice site changes by these in silico splice site prediction tools, for each disease‐causing or neutral variant, we recorded their effect on corresponding natural splice sites by recording wild‐type and variant prediction scores (Table S2). We also recorded any cryptic sites that the variant created or strengthened significantly and are comparable to or stronger than their corresponding native site. For nucleotide substitutions located within consensus splice site sequences, 10% reduction in score is generally interpreted to affect splicing (Tang, Prosser, & Love, 2016). Whereas, for those located outside the consensus splice site sequences, no clear cutoff values were established. The context‐based outcome of splicing dysregulation by variants located outside consensus splice site sequences in our study also underlined the difficulty to establish threshold score changes for assessing such variants by in silico splice site tools. For the current study, disease‐causing or neutral variants were designated to affect splicing if they disrupted a native splice site (≥10% score decrease) or activated/created a strong cryptic site (≥90% of corresponding native site score). The predictions of creation/activation of cryptic acceptor sites in exon 1 and cryptic donor sites in exon 8 were not considered*.*

### 2.3 In silico analysis using SRE prediction tools

To study the effect of *F9* exonic disease‐causing and neutral variants on SREs, we employed ESRseq, HEXplorer, and EX‐SKIP prediction tools*.* ESRseq assigned scores to all possible hexamer sequences and designated them as enhancers or silencers based on their effect on splicing in minigene experiments performed in HEK293tTA cells. The effect of variants on SREs, thereby on splicing, is predicted by calculating the net ESRseq score change of hexamer sequences overlapping the variants (Ke et al., 2011). HEXplorer also assigned scores (Z_EI scores) to hexamer sequences, but the difference from ESRseq is that calculation of scores is based on hexamer frequencies in 100 nucleotides of exonic and intronic sequences flanking the known 5′splice sites. Similar to ESRseq, HEXplorer also predicts the effect of variants on splicing by calculating the net change in hexamer scores affected by variants (Erkelenz et al., 2014). ESRseq and HEXplorer scores varied significantly in the absolute values assigned to the hexamer sequences. However, for both tools, a net negative score would indicate the disruption of ESEs and/or creation of ESSs leading to exon skipping. EX‐SKIP tool (http://ex-skip.img.cas.cz/) combines the predictions of several individual SRE prediction tools (PESE and PESS, FAS‐ESS, RESCUE‐ESE, EIE and IIEs, NI‐ESE, and NIESS) for the presence of ESEs and ESSs and assigns scores based on their relative density (Raponi et al., 2011). Further, a ratio of ESS/ESE scores of variant over wild‐type sequence (EX‐SKIP ratio) is calculated and numbers >1 are considered predictive of increased exon skipping.

### 2.4 In vitro minigene assays

In vitro evaluation of selected *F9* disease‐causing and neutral variants for their effect on splicing was performed using minigene reporter constructs generated from pTBNde(min) plasmid (Pagani et al., 2003). For the generation of reporter constructs, wild‐type or variant *F9* exon sequences along with ~300 bp flanking intron sequences were directionally cloned. Transfection experiments were performed in HEK293 cells. All minigene reporters were transfected simultaneously using Lipofectamine 3000 reagent (Thermo Fisher Scientific, Waltham, MA). Total RNA was isolated 24 hr posttransfection using RNeasy Plus Mini Kit (Qiagen, Germantown, MD) and reverse transcription was performed using High‐Capacity cDNA Reverse Transcription Kit (Thermo Fisher Scientific). PCR amplification was performed with Platinum Hot Start PCR Master Mix (Thermo Fisher Scientific) using Alfa2‐3 forward primer; 5′‐CAACTTCAAGCTCCTAAGCCACTGC‐3′ and Bra2 reverse primer; 5′‐GTCACCAGGAAGTTGGTTAAATCA‐3′ (Fernandez Alanis et al., 2012). PCR reactions were limited to 25 cycles to stop the reaction in exponential phase of amplification. To visualize the resulting DNA products, PCR reactions were run on 3% agarose gels, stained with ethidium bromide and imaged with UVP BioDoc‐It Imaging Systems (Analytic Jena, Beverly, MA). DNA band intensities were quantified with ImageJ software (NIH, Bethesda, MD). The exon skipping induced by variants in this assay was calculated as percentage of skipped transcripts among total transcripts. Repeat transfection experiments were performed to confirm the consistency of results. Selected DNA products were gel‐purified using QIAquick Gel Extraction Kit (Qiagen) and sequenced at Facility for Biotechnology Resources, US Food and Drug Administration (Silver Spring, MD).

### 2.5 In vitro translation assay

The effect of c.484C>A variant (p.R162R) on FIX global translation rates was assessed by performing in vitro translation assay. Assay was performed with Rabbit Reticulocyte Lysate (RRL) cell‐free system (Promega) supplemented with calf liver tRNAs as described earlier (Simhadri et al., 2017).

## 3 RESULTS

### 3.1 In silico analysis using splice site tools predicted high number of F9 exonic point variants to induce splicing dysregulation

We employed NNSplice, MES, SSF‐like, and HSF tools to assess the effect of exonic disease‐causing and neutral variants on disruption of native splice sites and/or activation/creation of cryptic splice sites. A total of 535 variants from our curated database (Table 1; Table S1) that included 409 disease‐causing non‐synonymous variants, six disease‐causing synonymous variants, and 120 neutral variants (64 non‐synonymous and 56 synonymous) were analyzed. We first assessed the strength of the native splice sites of all *F9* exons predicted by the tools (Table 2). Our analysis showed that NNsplice and MES predicted a weak acceptor site for exon 4 and weak donor site for exon 5. NNsplice assigns a score of 0 to 1 for native splice sites. For exon 4 acceptor site, NNsplice reported a score of 0.22 against an average score of 0.97 (±0.03) for remaining exons. Similarly, for exon 5 donor site, NNsplice reported a score of 0.21 against an average score of 0.93 (±0.09) for other exons. MES assigns a score of 0 to 12 for native splice sites. For exon 4 acceptor site, MES reported a score of 5.14 against an average score of 8.75 (±1.23) for remaining exons. Similarly, for exon 5 donor site, MES reported a score of 3 against an average score of 8.52 (±0.74) for other exons. SSF‐like and HSF tools, which assign a score of 0–100 for splice sites reported relatively lower scores for acceptor and donor site of exon 4 and 5, respectively, but difference in scores compared to other native sites was relatively low (Table 2). An additional in silico splice site tool, alternative splice site predictor (ASSP) (http://wangcomputing.com/assp/) also reported relatively lower scores for acceptor site of exon 4 (2.56 vs. 9.9 ± 1.50 for other corresponding sites) and donor site of exon 5 (6.49 vs. 11.84 ± 1.17 for other corresponding sites), suggesting that these sites are possibly weak and less conserved.

**Table 2:** In silico prediction of strength of *F9* native splice sites

Exon	Acceptor sites				Donor sites			
	NNSplice (0–1)^a^	MaxEntScan (0–12)	SpliceSiteFinder‐like (0–100)	Human Splicing Finder (0–100)	NNSplice (0–1)	MaxEntScan (0–12)	SpliceSiteFinder‐like (0–100)	Human Splicing Finder (0–100)
1	N/A^b^	N/A	N/A	N/A	0.83	7.44	79.95	86.17
2	0.94	7.6	88.74	88.6	0.97	8.34	74.28	82.56
3	1	9.07	88.53	82.8	0.99	8.72	81.58	91.09
4	0.22	5.14	67.13	78.45	1	9.66	95.87	98.02
5	0.94	9.23	86.18	82.9	0.21	3	72.4	80.32
6	0.99	9.94	85.79	81.29	0.79	8.17	76.82	78.88
7	0.99	9.75	97.75	93.79	0.99	8.76	87.85	87.83
8	0.93	6.88	91.13	82.61	N/A^c^	N/A	N/A	N/A

a Numbers in parenthesis indicate the range of scores assigned to splice sites by individual tools. Lower number indicates weak or less conserved splice site and higher number indicates strong or highly conserved splice site.

b Exon 1 lacks natural acceptor site. Hence, no score was calculated and N/A (not applicable) was shown.

c Exon 8 lacks natural donor site. Hence, no score was calculated and N/A was shown.

**Figure 1:**
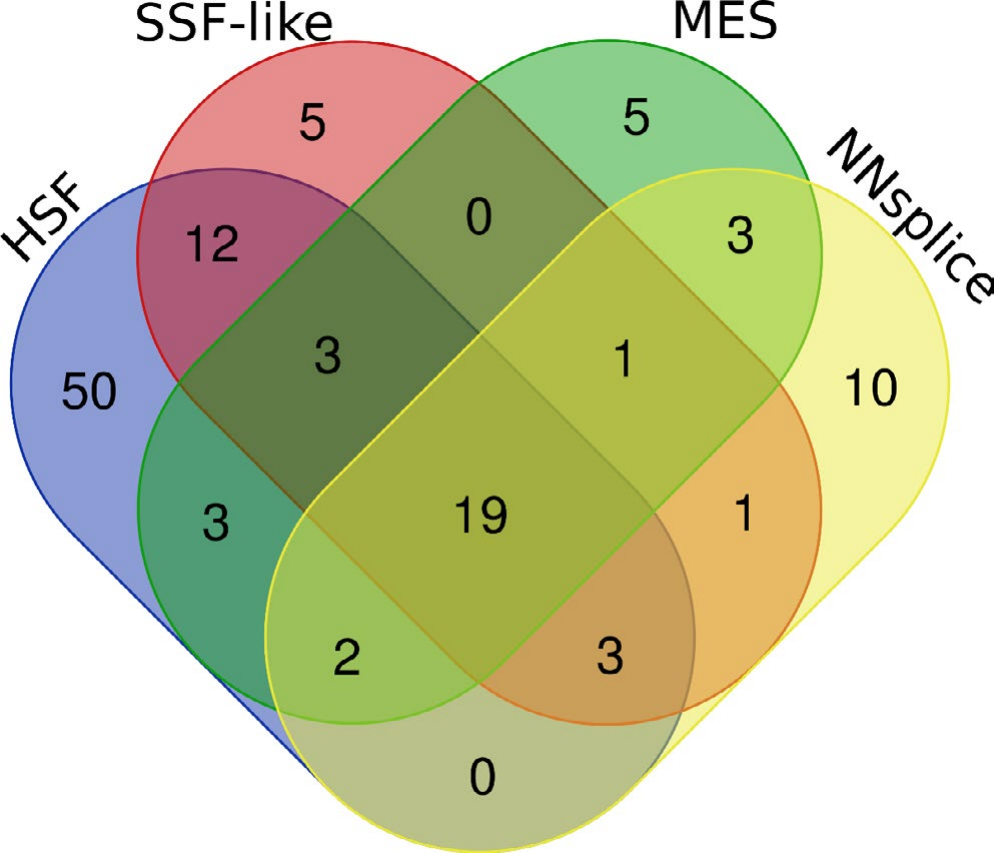
*F9* exonic variants predicted to affect splicing by in silico splice site tools. Venn diagram shows the overlap between *F9* exonic variants predicted to affect splicing by in silico splice site prediction tools employed in this study; NNsplice, MaxEntScan (MES), SpliceSiteFinder‐like (SSF‐like), and Human Splicing Finder (HSF)

**Figure 2:**
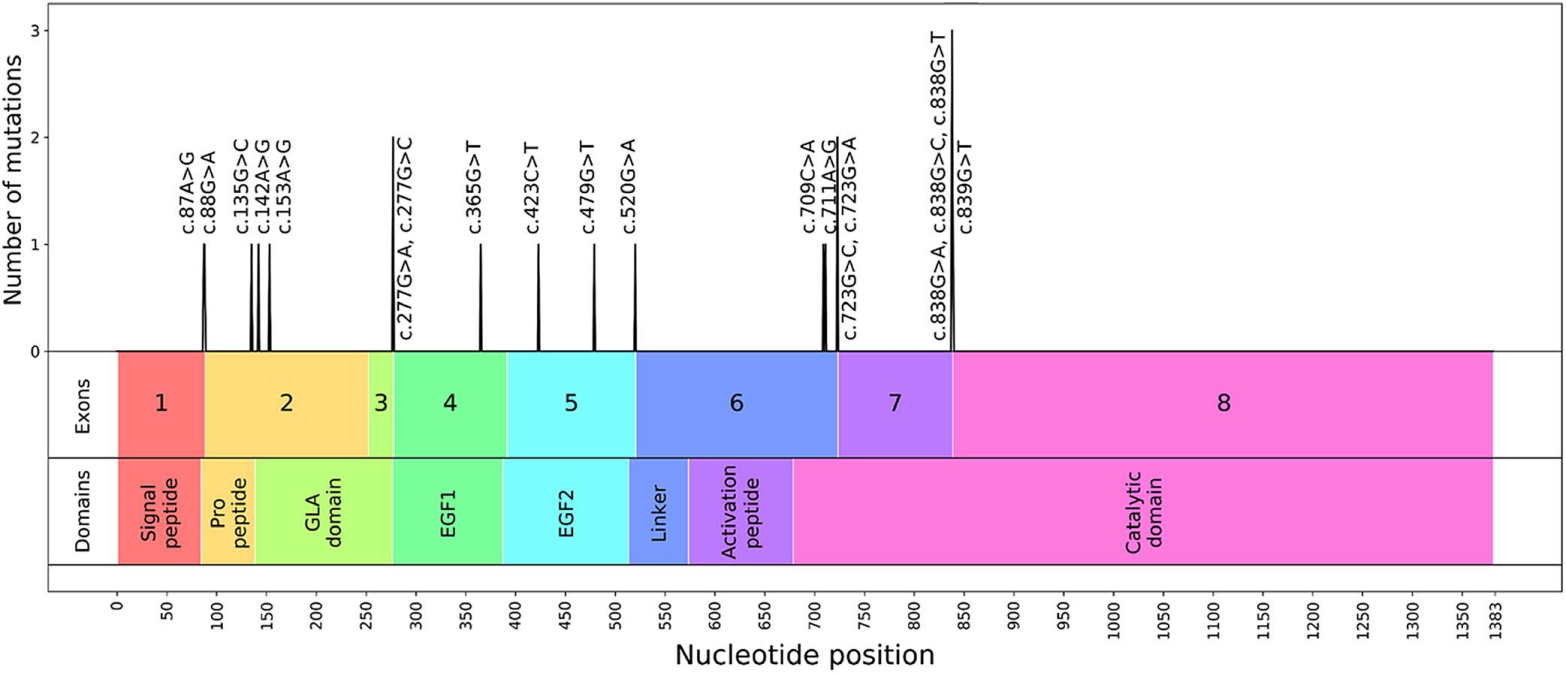
Location of *F9* exonic splicing variants predicted to affect splicing by all in silico splice site tools. Image shows the location of *F9* exonic variants that were predicted to affect splicing by all four in silico splice site tools (NNsplice, MaxEntScan, SpliceSiteFinder‐like, and Human Splicing Finder). Bars indicate the number of variants identified at each location

In our study, as previously described in the Materials and Methods section, variants were considered to affect splicing upon weakening of native site (≥10% score decrease) or activation/creation of a comparably strong cryptic site (≥90% of native score). Complete details of splice site score changes reported by in silico splice site prediction tools for *F9* exonic disease‐causing and neutral variants analyzed in this study are provided in Table S2. Using these criteria mentioned above, NNsplice, MES, SSF‐like, and HSF tools predicted 39, 36, 44, and 89 variants, respectively, to affect splicing. Classification of the variants predicted to affect splicing by variant type (non‐synonymous, synonymous, and neutral) and splicing dysregulation type (affecting native site or cryptic site) is provided in Tables 3 and 4, respectively. Among the four tools, HSF predicted higher number of variants to affect splicing, which was primarily due to the prediction of higher number of activation/creation of cryptic acceptor sites (Table 4). Further examination of the variants predicted to affect splicing revealed that 19 variants were predicted to affect splicing by all four tools and 28 variants were predicted to affect splicing by at least three tools (Figure 1). Commonly predicted variants by all tools, included 14 non‐synonymous variants, four synonymous variants, and one neutral variant. Of these, nine non‐synonymous and two synonymous variants disrupted native consensus splice sequences in exons (first and last two nucleotides of the exon; Figure 2). Of the six disease‐causing synonymous variants analyzed in this study, four were predicted to affect splicing by all four tools and one was predicted to affect splicing by three tools except HSF. One synonymous variant (c.484C>A) was not predicted to affect splicing by any of the tools.

**Table 3:** Classification of *F9* exonic disease‐causing and neutral variants predicted to affect splicing by in silico splice site tools based on variant type

Variant type	Number of variants analyzed	NNSplice	MaxEntScan	SpliceSiteFinder‐like	Human Splicing Finder
Disease‐causing non‐synonymous variants	409	29	27	35	73
Disease‐causing synonymous variants	6	5	5	5	4
Neutral non‐synonymous variants	64	1	1	1	4
Neutral synonymous variants	56	4	3	3	8
Total	535	39	36	44	89

**Table 4:** Classification of *F9* exonic disease‐causing and neutral variants predicted to affect splicing by in silico splice site tools based on splicing dysregulation mechanism

Mechanism	NNSplice	MaxEntScan	SpliceSiteFinder‐like	Human Splicing Finder
Disruption of native acceptor site	2	2	1	1
Disruption of native donor site	13	13	10	10
Activation/creation of cryptic acceptor site	10	10	17	60
Activation/creation of cryptic donor site	14	11	16	18
Total	39	36	44	89

### 3.2 In vitro minigene assays of selected variants revealed variable outcome of splicing dysregulation

**Figure 3:**
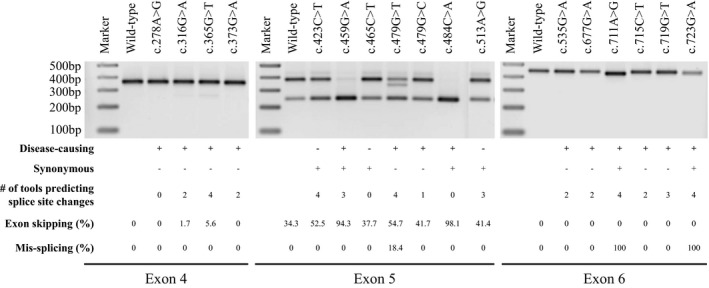
In vitro minigene analysis of select *F9* variants in exons 4, 5, and 6. Representative agarose gel image showing the PCR amplification products of the studied *F9* variants. Reporter minigene constructs were transfected into HEK293 cells and splicing pattern was assessed by PCR amplification of cDNA generated from total RNA. Expected sizes of PCR amplified products for exon 4, 5, and 6 reporter constructs were 360, 375, and 449 bp, respectively, in the event of normal splicing (exon inclusion) and 246 bp in the event of exon skipping. Exon skipping induced by individual variants was calculated using ImageJ software and numbers were shown in Table 5. The characteristics of tested variants with regard to disease‐causing (+) or not (−), synonymous (+) or not (−), number of tools (out of four in silico splice site tools; NNsplice, MaxEntScan, SpliceSiteFinder‐like, and Human Splicing Finder) that predicted splice site dysregulation, the extent of exon skipping and/or mis‐splicing in minigene assays, and their exonic locations are indicated below

To validate the predictions of the in silico splice site tools, in vitro minigene assays were performed for selected variants. Details of variants selected for this assay, in silico predictions by splice site tools, and results from minigene assays are shown in Table 5 and Figure 3. Variants included in this analysis were primarily selected from exons 4, 5, and 6 which offer varied contexts of native splice sites, wherein exon 4 has weak acceptor site, exon 5 has weak donor site, and exon 6 has no weak native splice sites. The selected mutations included all: synonymous, non‐synonymous, and neutral variants. In addition, we included variants that have variable effects on splice sites (disrupting native splice sites, activating/creating cryptic sites, or no effect).

**Table 5:** *F9* exonic variants assessed by minigene assays and in silico predictions of their effect on splicing regulation

Nucleotide change	Exon	Mutation type	Disease‐causing	Relative distance to		NN splice	MaxEnt Scan	Human Splicing Finder	SpliceSiteFinder‐like	ΔESRseq score	ΔHEXplorer score	EX‐SKIP ratio	Exon skipping based on minigene assay (%)^h^	Comments
				Acceptor site	Donor site									
c.278A>G	4	Non‐syn^a^	Yes	0	113	NA^c^	NA	NA	NA				0	Increases native acceptor site score
c.316G>A	4	Non‐syn.	Yes	38	75	ne^d^	ne	CA^e^	CA	−0.764	30.14	0.966	1.74 ± 0.52	
c.365G>T	4	Non‐syn.	Yes	87	26	CA	CA	CA	CA	−2.395	−105.18	1.241	5.59 ± 0.29	
c.373G>A	4	Non‐syn.	Yes	95	18	ne	ne	CA	CA	0.22	3.2	0.983	0	
c.423C>T	5	Syn.^b^	No	31	97	CD^f^	CD	CD	CD	−0.894	−43.84	1.058	52.5 ± 0.24	Wild‐type exon 5 sequence showed 34.31 ± 1.15% exon skipping
c.459G>A	5	Syn.	Yes	67	61	CD	CD	ne	CD	−0.962	−30.13	1.115	94.34 ± 0.29	
c.465C>T	5	Syn.	No	73	55	ne	ne	ne	ne	0.529	−42.25	1.327	37.66 ± 1.18	
c.479G>T	5	Non‐syn.	Yes	87	41	CD	CD	CD	CD	−1.356	−15.42	1.173	54.73 ± 1.03	18.42 ± 0.77% mis‐splicing
c.479G>C	5	Non‐syn.	Yes	87	41	ne	ne	ne	CD	0.255	18.22	0.981	41.65 ± 0.88	
c.484C>A	5	Syn.	Yes	92	36	ne	ne	ne	ne	−2.202	−79.82	1.058	98.1 ± 0.40	
c.513A>G	5	Syn.	No	121	7	CD	CD	CD	ne	−1.375	−30.58	1.096	41.35 ± 0.21	
c.535G>A	6	Non‐syn.	Yes	14	188	ne	ne	CA	CA	−2.96	−46.36	1.035	0	
c.677G>A	6	Non‐syn.	Yes	156	46	ne	ne	CA	CA	−2.012	−13.58	0.965	0	
c.711A>G	6	Syn.	Yes	190	12	CD	CD	CD	CD	−0.862	−5	1	100% mis‐splicing	
c.715C>T	6	Non‐syn.	Yes	194	8	ne	ne	CA	CA	0.812	−18.65	1.053	0	
c.719G>T	6	Non‐syn.	Yes	198	4	CA	ne	CA	CA	−1.301	17.68	1.035	0	
c.723G>A	6	Syn.	Yes	202	0	ND^g^	ND	ND + CD	ND + CD				100% mis‐splicing	Decreases native donor site score

a Non‐synonymous.

b Synonymous.

c Affects native acceptor site.

d No predicted effect on splicing.

e Activation or creation of cryptic acceptor site.

f Activation or creation of cryptic donor site.

g Affects native donor site.

h Numbers indicate exon skipping percentage (±*SD*) for each mutation (Figure 3).

In exon 4, we evaluated four disease‐causing non‐synonymous variants: c.278A>G, c.316G>A, c.365G>T, and c.373G>A in comparison with wild‐type exon 4 sequence. In the minigene assays, exon 4 reporter constructs should yield a 360 bp PCR product upon normal splicing (exon inclusion) and 246 bp PCR product in the event of exon skipping. In our study, exon 4 wild‐type sequence and c.278A>G variant, that was predicted to increase native acceptor site strength and positively influence splicing, showed normal splicing with 100% exon inclusion. C.316G>A and c.373G>A variants, that were predicted to create a new cryptic acceptor site of significant strength by SSF‐like and HSF tools, showed 0 and 1.74% of exon skipping, respectively. C.365G>T variant, that was predicted to activate a cryptic acceptor site by all four tools, showed 5.59% exon skipping.

In exon 5, we evaluated the following variants: c.423C>T, c.459G>A, c.465C>T, c.479G>T, c.479G>C, c.484C>A, and c.513A>G in comparison with wild‐type exon 5 sequence. In the minigene assays, exon 5 reporter constructs should yield a 375 bp PCR product upon normal splicing and 246 bp PCR product in the event of exon skipping. In our study, exon 5 wild‐type construct showed 34.31 exon skipping (Figure 3). This result was expected as exon skipping of the wild‐type exon 5 sequence in both in vitro minigene assays and in vivo (human liver tissue) was reported in earlier studies (Balestra et al., 2016; Tajnik et al., 2016). The exon skipping observed for the wild‐type sequence is likely due to a weak donor site as predicted by the tools. In exon 5, c.459G>A and c.484C>A are disease‐causing synonymous variants. Of these, c.459G>A was predicted to activate a new cryptic donor site by three tools, excluding HSF, whereas c.484C>A was not predicted to affect splicing by all four tools. In our study, both c.459G>A and c.484C>A variants induced significant exon skipping (94.34% and 98.1%, respectively). C.423C>T and c.513A>G are synonymous neutral variants that were predicted to create a cryptic donor site by majority of tools. Interestingly, neutral variants, c.423C>T and c.513A>G, showed higher exon skipping than wild‐type sequence with rates of 52.5 and 41.35%, respectively. c.465C>T is also a synonymous neutral variant that was not predicted to affect splicing by all four tools. This variant showed 37.66% exon skipping which was similar to exon skipping observed for the wild‐type sequence. Finally, c.479G>T and c.479G>C are non‐synonymous disease‐causing variants. Of these, c.479G>T was predicted to create a new cryptic donor site by all four tools, whereas c.479G>C was predicted to create a new cryptic donor site by SSF‐like tool only. In the minigene assay, c.479G>T showed significant exon skipping (54.73%) as well as mis‐splicing (18.42%) with a loss of 43 nucleotides in exon that was confirmed by subsequent sequencing of mis‐spliced product (Table 6). On the other hand, c.479G>C showed 41.65% exon skipping.

**Table 6:** *F9* variants showing mis‐splicing in minigene assay

Variant	Exon location	Native donor site sequence	Cryptic donor site sequence	Native exon length	Mis‐spliced exon length	Predicted cryptic donor site score over native donor site (% difference)			
						NNsplice	MES	HSF	SSF‐like
c.479G>T	5	CAGgtcataa	GAGgtatatc	129	86	304.7	143.3	−3.8	3.1
c.711A>G	6	CAGgtacttt	CAGgtcagtt	203	186	21.5	9.4	17.3	20.4
c.723G>A	6	CAGgtacttt	TTGgcaagta	203	197	Cryptic site not detected	Cryptic site not detected	−2.7	30.2

Abbreviations: HSF, Human Splicing Finder; MES, MaxEntScan; SSF, SpliceSiteFinder.

In exon 6, we evaluated the following variants: c.535G>A, c.677G>A, c.711A>G, c.715C>T, c.719G>T, and c.723G>A in comparison with wild‐type exon 6 sequence. In the minigene assays, exon 6 reporter constructs should yield a 449 bp PCR product upon normal splicing and 246 bp PCR product in the event of exon skipping. Of the variants analyzed, c.535G>A, c.677G>A, c.715C>T, and c.719G>T are non‐synonymous disease‐causing variants that were predicted to create/activate cryptic acceptor sites predominantly by SSF‐like and HSF tools. In minigene assay, these variants showed normal splicing with 100% exon inclusion similar to wild‐type sequence. In exon 6, c.711A>G and c.723G>A are synonymous disease‐causing variants. Of these, c.711A>G was predicted to activate a new cryptic donor site by all four tools. In our study, this variant induced mis‐splicing with the loss of 17 nucleotides in the exon (Table 6). The second synonymous variant, c.723G>A, disrupts natural donor site consensus sequence and was predicted to significantly lower its strength by all four tools. In addition, SSF‐like and HSF tools predicted this variant to create new cryptic donor site 4 nucleotides upstream of native donor site (Table 6). In the minigene assay, c.723G>A induced mis‐splicing as predicted by SSF‐like and HSF tools. Mis‐splicing induced by both c.711A>G and c.723G>A variants was confirmed by subsequent sequencing of PCR products.

### 3.3 In silico analysis using SRE prediction tools revealed the contribution of SRE disruption to splicing dysregulation

Results from minigene assays showed that for some variants that were predicted to activate/create a cryptic acceptor site or donor site (e.g., c.365G>T, c.423C>T, and c.459G>A), the predominant effect on splicing was exon skipping rather than mis‐splicing from the use of predicted cryptic splice sites. Also, c.484C>A, a disease‐causing synonymous variant that was not predicted to effect splicing by all splice site prediction tools showed significant exon skipping. These results suggested a possible role of SREs in the dysregulation of splicing by these variants. To assess the dysregulation of SREs by the exonic variants under study, we calculated the score changes for variant sites in comparison with native sequence using ESRseq, HEXplorer methods, and the EX‐SKIP tool (Table 5). For variant sequences, a net negative score change calculated by ESRseq and HEXplorer methods and a ratio of >1 by EX‐SKIP tool indicates the disruption of ESEs and/or creation of ESSs and predicts increased exon skipping. We excluded c.278A>G and c.723G>A variants from this analysis due to their location within the consensus splice site sequences. Individually, for variants c.365G>T, c.423C>T, c.459G>A, c.479G>T, and c.484C>A which showed relatively higher exon skipping in their respective exons, the numbers reported by all three methods suggested increased exon skipping. Also, for the c.373G>A variant in exon 4 that showed no exon skipping, numbers reported by all tools were in agreement suggesting a lack of dysregulation of SREs. For rest of the variants, predictions were inconsistent, especially for exon 6 variants. Among the individual tools, overall score changes calculated by ESRseq method were in general agreement with the increased exon skipping observed for variants in exons 4 and 5. This finding need to be viewed with the consideration that our dataset, which was selected to represent various criteria of variants for minigene assay, was small and may not be suitable for measuring overall tendency or statistical association. Additionally, some variants (c.479G>T, c.711A>G, and c.723G>A) were involved in additional splicing dysregulation of activation/creation of cryptic splice sites and subsequent mis‐splicing.

### 3.4 In vitro translation assay revealed additional disease induction mechanism of c.484C>A synonymous variant

**Figure 4:**
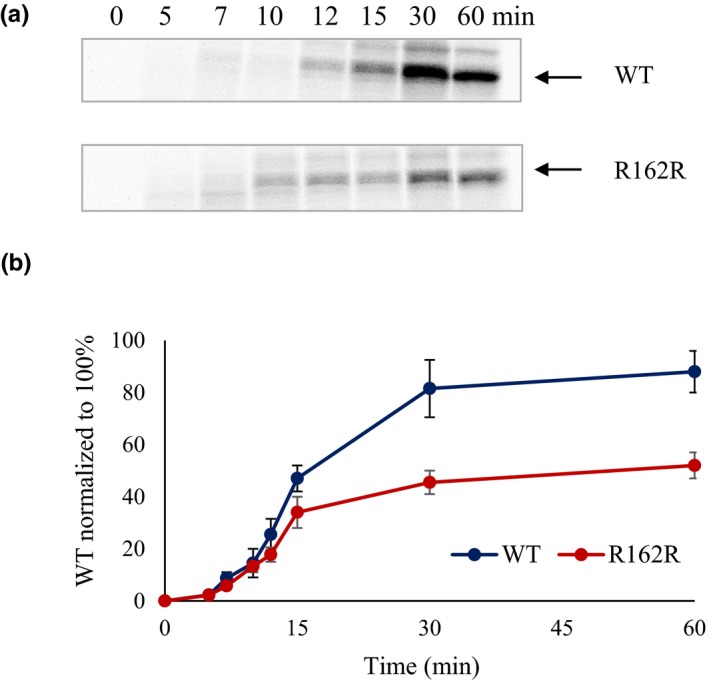
In vitro translation analysis of c.484C>A (p.R162R) variant. Panel (a) shows the representative autoradiogram of FIX wild‐type (WT) and FIX p.R162R (c.484C>A) in vitro translation products. Panel (b) shows the graphical representation of intensities of in vitro translation products. Error bars represent *SD* values

In addition to splicing, synonymous variants were reported to induce disease phenotype through altered mRNA and protein characteristics (Hunt et al., 2014). The lack of predicted effect of c.484C>A mutation on splicing by in silico splice site tools in our preliminary analysis prompted us to assess its impact on translation rate using in vitro translation assay. In this assay, c.484C>A showed significant reduction in the translation rate of variant *F9* mRNA (Figure 4).

## 4 DISCUSSION

In this study, we analyzed a curated dataset of 535 *F9* exonic variants including 415 disease‐causing (409 non‐synonymous and six synonymous) and 120 neutral variants (64 non‐synonymous and 56 synonymous) for their effect on splicing using in silico splice site prediction tools and further evaluated selected variants using in vitro minigene assays and in silico SRE prediction tools.

Analysis of strength of natural splice sites in the eight exons of the *F9* gene indicated that exon 4 and 5 carry weak acceptor and donor site, respectively (Table 2). The scores assigned by NNsplice, MES, and ASSP tools to these sites were lower compared to other corresponding *F9* splice sites. The in silico splice site tools employed in this study, NNsplice, MES, SSF‐like, and HSF, predicted 39, 36, 44, and 89 variants, respectively, to affect splicing via disruption of native splice sites or creation/activation of cryptic splice sites (Tables 3 and 4). Of the 19 variants that were predicted by all four tools, 11 were located within the consensus splice sequences in exons (first and last two of the exonic nucleotides; Figure 2) affecting native splice sites. The splice site tools perform relatively better within consensus splice sites when compared to distantly located variants (Jian, Boerwinkle, & Liu, 2014). A total of 15 variants in consensus splice sites were analyzed in this study. The remaining four variants in the consensus sequences were not predicted to affect splicing by NNsplice, SSF‐like, and HSF tools as score changes were lower than 10%. These results suggested that the prediction tools are mostly in agreement with variants located in consensus splice site sequences. Also, these variants are more likely to be involved in splicing events.

We evaluated a selected list of variants including four synonymous, 10 non‐synonymous and three neutral variants from exons 4, 5, and 6 using in vitro minigene assays (Table 5). This carefully chosen set of variants, which represent exons of varied natural splice site strength, both disease‐causing and neutral variants and variable mechanism of splicing dysregulation, showed interesting outcomes in the minigene assays. All the synonymous disease‐causing variants studied by minigene assay in this study (c.459G>A and c.484C>A in exon 5 and c.711A>G and c.723G>A in exon 6) showed splicing dysregulation. Of these, c.484C>A was not predicted to affect splicing by the splice site prediction tools, but further examination by SRE prediction tools suggested disruption of ESEs and/or creation of ESSs and increased potential of exon skipping. Results from the minigene assay for c.484C>A were in agreement with this last prediction. The c.723G>A variant alters the last nucleotide of exon 6 disrupting native 5′ss consensus sequence. All four splice site prediction tools predicted the disruption of native donor site and reported lower scores. SSF‐like and HSF tools have additionally predicted the activation of an upstream (four nucleotides) cryptic donor site. This activated cryptic donor site involved noncanonical splice site pair GC–AG which was appreciated to occur at a very low frequency (<1%) compared to commonly used (>99%) canonical splice site pair GT–AG (Burset, Seledtsov, & Solovyev, 2000). The failure of NNsplice and MES tools in identification of the cryptic donor site activation by c.723G>A was probably due to noninclusion of noncanonical splice site pairs in their prediction model (Reese et al., 1997; Yeo & Burge, 2004). Two other synonymous variants in our curated database were also predicted to affect splicing either by disruption of consensus splice site sequence (c.87A>G) or activation of a cryptic donor site with a score higher than native donor site (c.153A>G). Overall, all six disease‐causing synonymous variants analyzed in this study seem to induce splicing dysregulation. These results further show that splicing dysregulation is a common disease‐inducing mechanism of synonymous variants (Hunt et al., 2014). However, growing literature evidence suggests that synonymous variants can cause disease phenotype by several other mechanisms including altered mRNA structure and stability, miRNA binding, and altered codon usage affecting protein translation and folding (Athey et al., 2017; Bartoszewski et al., 2010; Hunt et al., 2014; Kimchi‐Sarfaty et al., 2006; Salzman & Weidhaas, 2011). Here, the c.484C>A synonymous variant showed significant reduction in the global translation rate compared to wild‐type (Figure 4) in in vitro translation assay. While the very high rate of exon skipping caused by this variant seems to be the major mechanism of disease induction, diminished translation rate of the mature mRNA likely has a compounding effect. Recently, the c.459G>A variant in *F9*, which showed dysregulation of splicing in our study and others (Tajnik et al., 2016), was reported to alter protein confirmation via altered translation kinetics and result in decreased specific activity of FIX (Simhadri et al., 2017) suggesting that alternative or concomitant mechanisms of disease induction for synonymous variants should not be ignored.

The non‐synonymous variants tested in minigene assays included four in exons 4 and 6 and two in exon 5. Except for the c.278A>G variant in exon 4, all other variants were located outside consensus splice site sequences. In our study, several of these non‐synonymous variants (e.g., c.316G>A and c.365G>T in exon 4, c.479G>T and c.479G>C in exon 5) showed increased exon skipping relative to respective wild‐type sequences. Interestingly, in addition to exon skipping, c.479G>T also induced mis‐splicing through activation of cryptic donor site. These results underscore how splicing dysregulation can contribute partially or wholly to disease phenotype induced by non‐synonymous variants. All the neutral variants tested by minigene assays were synonymous and located in exon 5. Of these, c.423C>T and c.513A>G variants showed increased exon skipping relative to wild‐type sequence. The partial dysregulation of splicing induced by these neutral variants may not be sufficient to lower the serum FIX levels (to <40%) to induce hemophilia B. Overall, results from our study show that *F9* exonic non‐synonymous and neutral variants located outside consensus splice site sequences can induce splicing dysregulation either through activation/creation of cryptic splice sites and/or disruption of SREs. This finding is in agreement with recent literature that reported the prevalence of exonic splicing variants with up to 7% of exonic variants being predicted to disrupt splicing (Caminsky et al., 2015; Soukarieh et al., 2016).

An important observation from the minigene assay data was that some of the variants that were predicted to activate a cryptic donor site showed mis‐splicing (c.479G>T, c.711A>G, and c.723G>A), whereas none of the variants that were predicted to activate a cryptic acceptor site showed mis‐splicing (c.365G>T, c.719G>T etc.). Branch point recognition plays an important role in acceptor site selection during pre‐mRNA splicing (Mercer et al., 2015). The splice site prediction tools typically model for consensus sequence of acceptor sites in isolation from branch point site and its adjacent polypyrimidine tract sequences, which makes predictions for cryptic acceptor sites less reliable. Results from minigene assays in our study were in agreement with this observation.

Additionally, variants in exons 4 and 5 readily induced exon skipping compared to exon 6 variants. Variants in exon 6 showed mis‐splicing or no effect on splicing. Furthermore, within exons 4 and 5, the level of exon skipping was higher for variants in exon 5 compared to exon 4. As mentioned, exon 4 and 5 seem to have weak acceptor and donor site, respectively. Previous studies suggested that SR proteins improve the inclusion of exons with weak splice sites through their action on ESEs and in the event of their disruption, exons with weak splice sites are more susceptible to splicing defects (Grodecka, Buratti, & Freiberger, 2017; Roca, Krainer, & Eperon, 2013). Although this is a small dataset, our results suggest that the exon skipping events observed for variants in exons 4 and 5 are a result of disruption of SREs in the context of weak splice sites.

In our study, in silico splice site prediction tools were predictive of the activation of cryptic donor sites by variants located outside consensus sequences in exon sequences (c.479G>T and c.711A>G). For some variants, the predicted cryptic splice sites did not translate into mis‐splicing and a subsection of these showed variable levels of exon skipping (e.g., c.365G>T and c.423C>T). For the variants that showed relatively higher levels of exon skipping (c.365G>T, c.423C>T, c.459G>A, c.479G>T, and c.484C>A), in silico SRE prediction tools predicted exon skipping due to disruption of ESEs and/or creation of ESSs. The exon skipping observed for c.484C>A seems to be clearly due to disruption of SREs as no cryptic splice site activation was predicted by splice site tools. However, for remaining variants, whether the observed exon skipping was primarily due to disruption of SREs or a combination of creation/activation of a competitive cryptic splice sites and disruption of SREs were not clear. Overall, our results indicate that splice site and SRE prediction tools used in tandem provide a better prediction of splicing dysregulation induced by exonic variants. However, as one would expect these tools were not always correctly predictive of the splicing dysregulation as we observed for several of the exon 6 variants. Also, the net outcome of splicing dysregulation differed for variants across exons and not always predictable by in silico tools as it will depend on several additional factors including location and strength of cryptic splice sites, strength of corresponding native splice sites, and density of SREs (Caminsky et al., 2015; Grodecka et al., 2017). In this regard, outcome of splicing dysregulation can be best assessed by performing minigene assay of variant in the context of native exon sequence.

In conclusion, our study suggests that splicing dysregulation may contribute to the pathogenicity of majority of synonymous variants and several non‐synonymous variants in *F9*. The in silico splice site and SRE prediction tools when used in tandem more accurately predicted spicing dysregulation induced by such variants. The net effect of point variants on splicing dysregulation is context dependent and can be difficult to predict by in silico prediction tools. When primary tissue samples are not available, as is often the case, minigene assays remain a straightforward approach to assess novel variants for splicing effects. While the findings in this work were drawn from studying variants in *F9*, we anticipate that these findings may be recapitulated upon careful analysis of variants in other genes as well.

## CONFLICT OF INTERESTS

None declared.

## DISCLAIMER

Our comments/our contributions are an informal communication and represent our best judgment. These comments do not bind or obligate FDA.
