# Supplementary material for: Splicing dysregulation contributes to the pathogenicity of several F9 exonic point variants
Source: Mol Genet Genomic Med. 2019 Jun 30;7(8):e840. doi: 10.1002/mgg3.840 (PMC6687662; doi:10.1002/mgg3.840)
Supplement: Supplementary file 3 [file MGG3-7-e840-s003.docx]

Table S1. List of *F9* exonic disease-causing and neutral variants analyzed in this study.

Table S2. Details of splice site score changes reported by *in silico* splice site prediction tools for *F9* exonic disease-causing and neutral variants analyzed in this study.
